# Movement Assessment Battery for Children-Second Edition (MABC2): Cross-Cultural Validity, Content Validity, and Interrater Reliability in Thai Children

**DOI:** 10.1155/2019/4086594

**Published:** 2019-12-13

**Authors:** Rujira Jaikaew, Nuntanee Satiansukpong

**Affiliations:** Department of Occupational Therapy, Faculty of Associated Medical Sciences, Chiang Mai University, 110 Intawaroroj Rd., Sripoom, Chiang Mai 50200, Thailand

## Abstract

**Introduction:**

The Movement Assessment Battery for Children-Second Edition (MABC2) is a standardized test for detecting children with movement difficulty. It was established and is used widely in Western countries. Studying cross-cultural validity and reliability was necessary before using the MABC2 with Thai children.

**Purposes:**

To study cross-cultural validity, content validity, and interrater reliability of the MABC2.

**Method:**

The MABC2-Age Band 2 (AB2: children aged 7-10 years) was translated into Thai from the source version of the MABC2 by using the following steps: forward translation, backward translation, panel discussion, and testing of the prefinal version of the Thai-MABC2-AB2. Five occupational therapists checked the content validity of the test. Twenty-nine children, aged 7-10 years, were examined by two testers in order to establish interrater reliability.

**Results:**

This cross-cultural study demonstrated validity in the Thai context. Content validity was good with an item-objective congruence (IOC) range from 0.73 to 0.95. The intraclass correlation coefficient (ICC) of interrater reliability ranged from 0.71 to 1.00.

**Conclusion:**

The Thai-MABC2-AB2 is a good fit for use in a clinical and Thai cultural setting. Interrater reliability was moderate to good, which meant results between testers were consistent.

## 1. Introduction

Motor skills are an important ability that have influence on the occupation of children. Children use movement to perform activities at home and school and in the community, such as dressing, eating, writing, participating in physical activities, and playing with friends in their free time. Children with movement difficulties may struggle in performing activities of daily living (ADL) and academic and social activities. The problems of movement difficulty might lead to secondary ones in children, for instance, low self-esteem, low self-confidence, anxiety, and social isolation [1]. Thus, early detection of movement difficulty is necessary in order to provide early intervention.

A standardized test is used to detect a certain area of difficulty among children, monitor a child's performance and developmental progress, and evaluate intervention programs. It also allows health professionals to speak the same language [2].

The Movement Assessment Battery for Children-Second Edition (MABC2) is a comprehensive measurement of motor skills and is a well-known standardized test for detecting movement difficulty in children. The test is used as a diagnostic tool for Developmental Coordination Disorder (DCD) in Western countries [3–7]. It is also used for detecting movement difficulty in Asian and South American countries, for instance, China, Japan, Taiwan, and Brazil [8–11]. According to the scoring system, the raw scores are converted to Standard Scores (SSs). SSs are converted to percentile ranks and then interpreted in terms of percentile equivalent: difficulty (at or below the 5th percentile), at risk (between the 6th and 15th percentile), and typical (above the 15th percentile) [12].

The MABC2 psychometric property studies have been increasing for over a decade. They reported that concurrent validity of the MABC2-Age Band 3, with the BOT-2, was good (*r* = 0.80) [13] and the MABC2-Age Band 1, with PDMS-2, was moderate (*r* = 0.63) [8]. Previous construct validity of the MABC2 in Greek, German, and Japanese studies showed equal validity to the original UK version [6, 9, 14].

The test showed consistent results for reliability. Test-retest reliability was good in both typical children (ICC = 0.83-0.98) [8] and children with DCD (ICC = 0.97) [10]. Internal consistency reliability was in an acceptable range (Cronbach's alpha = 0.50-0.70) [8, 9, 14]. Intrarater reliability was good, showing intraclass correlation coefficient (ICC) values of 0.88 [11]. Furthermore, the MABC2 was found to be sensitive and specific in screening movement difficulty in children [10]. A study of 43 Brazilian children, aged 6-11 years, recorded a good interrater reliability (ICC = 0.86 to 0.99) [11]. The other study on 30 children, aged 7-9 years, found poor to moderate interrater reliability (ICC = 0.39 to 0.68) [3]. Most of the above information supported that the MABC2 was suitable for detecting movement difficulty in children.

Psychometric properties are very important when it comes to clinicians choosing a tool for clinical setting and research. Validity is a crucial property that measures the purpose of the test [15]. Reliability is the consistency of measurement at different occasions or in a variety of conditions. When using the tests outside the original country, it is important to realize that they are potential influence on cultural and geographical difference. Thus, a psychometric property study is needed [16].

The advantage of the MABC2 is its age range for children of 3-16 years, in comparison to the Peabody Developmental Motor Scales-Second Edition (PDMS-2) and the Bruininks-Oseretsky Test of Motor Proficiency-Second Edition (BOT-2), which use a narrow age range of 0-5 years and wider age range of 4-21 years, respectively. The MABC2 is less time-consuming (20-40 minutes) compared to PDMS-2 (45-60 minutes) and BOT-2 (40-60 minutes). Furthermore, the test items are real-life activities and appropriate for each child's age.

To date, there has been no standardized test for detecting movement difficulty in Thai children. A cross-cultural validity study is an important part in preparing a test used in a different context from the original one. The content of the test should be validated in the language of the target country, and the translated version should be examined for its reliability. Therefore, the purposes of this study were to examine the cross-cultural validity of the MABC2 in the Thai context, investigate content validity, and research interrater reliability.

## 2. Methods

### 2.1. Participants

Five occupational therapists, with 10 years of experience in the pediatric field, were recruited to assess five typical children for testing the prefinal version of the MABC2, Thai version. Two more occupational therapists, with 2 years of experience in the pediatric field, were recruited as testers for the interrater reliability study.

Five typical children were recruited for testing the prefinal version of the Thai-MABC2-Age Band 2 (AB2). Thirty typical Thai children from a primary school in Muang district, Chiang Mai, Thailand, were recruited for this interrater reliability study. The inclusion criteria comprised (1) children aged 7 years and 0 months to 10 years and 11 months and (2) no physical limitations. The exclusion criterion consisted of (1) inability to follow commands and (2) inability to complete the test items in the Thai-MABC2-AB2. All of the participants and their parents signed a consent form before testing. The study protocol was approved by the Ethics Committee, Faculty of Associated Medical Sciences, Chiang Mai University, Thailand (AMSEC-60EX-042).

### 2.2. Instrument

The MABC2-AB2 was translated into the Thai language, permitted by The Pearson Corporation on 19 December 2017. The test comprises 3 subtests: (1) Manual Dexterity, (2) Aiming and Catching, and (3) Balance. The children were asked to perform 3 activities in the Manual Dexterity subtest (Placing Pegs, Threading Lace, and Drawing a Trail), 2 activities in the Aiming and Catching subtest (Catching with Two Hands and Throwing a Beanbag onto a Mat), and 3 activities in the Balance subtest (One-Balance Board, Walking Heel-to-Toe Forwards, and Hopping on Mats). All the test item activities took 20 to 30 minutes.

### 2.3. Procedures

The cross-cultural validity study followed the six steps of the Beaton protocol for translation from one language to another [17].

Step 1. The first step is forward translation from English to the Thai language. This step was performed by two translators, one with a medical background and the other with a nonmedical one. They independently translated the source version of the MABC2 into the Thai versions
Step 2. The second one is the synthesis of the translations. The two Thai versions (one from each translator) were merged into one following the discussion with the two translators and two researchers
Step 3. Backward translations, in which the tests were translated back into English, were completed by two translators, one with a psychology background and the other with a nonpsychology one
Step 4. A panel discussion was organized between two forward translators and two backward translators, an occupational therapist, a language specialist, and a researcher in order to check language equivalence between the source version of the MABC2 and the Thai-MABC2-AB2.
Step 5. The fifth step is testing the prefinal version. Five occupational therapists assessed 5 healthy children separately using the prefinal version
Step 6. The last one is the submission of all the translated versions to the Pearson Corporation

Next, content validity was examined. Five therapists checked for language clarity by using item-objective congruence (IOC), which followed the values of (i) +1 = clear, (ii) 0 = uncertain, and (iii) -1 = unclear. Language pertinence was checked by IOC, which followed the values of (i) +1 = appropriate, (ii) 0 = uncertain, and (iii) -1 = inappropriate. The IOC of each item was calculated by dividing the total scores by five.

Interrater reliability was established as follows: two testers were trained by a researcher to administer the Thai-MABC2-AB2. While testing, one tester administered the test, while the other one observed. Both testers scored each child independently. The scores between the two testers were computed using the Intraclass Correlation Coefficient (ICC).

### 2.4. Data Analyses

All data were analyzed using the SPSS program for Windows, version 17. Descriptive statistics was used. Content validity was analyzed using the IOC index. IOC values below 0.5 and above 0.5 were unacceptable and acceptable, respectively [18]. Raw scores were used to compute ICC for interrater reliability. Based on the 95% of Confident Interval (CI) of ICC estimate, values below 0.5, between 0.51 and 0.75, and above 0.75 were poor, moderate, and good reliability, respectively [19].

## 3. Results

### 3.1. Cross-Cultural Validity Study

Regarding the translation processes, forward translation, synthesis of the translations, and back translation showed no major difference between English and Thai meaning, though different words, phrases, and sentences were used. These Thai words and sentences were adjusted in the panel discussion among the experts in order to get clearer meaning.

Regarding the test of the prefinal version, the participants were able to follow most of the instructions. Five testers administered and demonstrated the same actions to the participants, which indicated that they had understood most of the instructions clearly. Only the Thai phrase in the Aiming and Catching subtest for “throwing overarm” was adjusted, because it has lead testers to demonstrate and administrate in the wrong action. Thus, the meaning of throwing overarm was clarified for both the testers and children in order that all testers understood and administrated in the same action.

### 3.2. Content Validity

The content validity ranged from 0.73 to 0.95 as shown in Table 1.

### 3.3. Interrater Reliability Study

One participant did not complete the test, due to the physical limitation of an ankle injury. Thus, the results of 29 typical children (14 boys and 15 girls; mean age = 9.08 years, standard deviation (SD) = 0.49) were used in this study. The interrater reliability of the Thai-MABC2-AB2 ranged from 0.71 to 1.00 as shown in Table 2.

## 4. Discussion

The MABC2 test is used worldwide to detect the movement difficulty in children. However, cross-cultural validity is necessary when using the test in a different context. Regarding cross-cultural validity of the Thai-MABC2-AB2, the Thai phrase of “throwing overarm” in the Aiming and Catching subtest was misunderstood by the testers. Some testers told the children to throw the ball up high underarm instead of throwing overarm. Therefore, this Thai phrase was adjusted so that all of the testers understood the same action. The test of the prefinal version was a significant step in completing cross-cultural validity and determining whether it is practical for testers. The content comparison between the Thai-MABC2-AB2 and the source version showed equivalence. Furthermore, the Thai children could complete the test with clear understanding because the activities used in the test's items were similar to the play activities in them. For example, the Hopping on Mats item is similar to playing Thai jumping, which is just like hopscotch, and the Threading Lace item is similar to threading leaves and flowers to make a garland for Thai children. The Thai-MABC2-AB2 showed good content validity and indicated that it can be used to test motor performance in Thai children.

The test was administered to 29 typical Thai children and scored by two Thai testers. The interrater reliability was moderate to good (ICC = 0.71-1.00). This indicated no significant difference of administration and scoring between testers. Most of the test's items, including Placing Pegs, Threading Lace, Catching with Two Hands, Throwing a Beanbag onto a Mat, One-Balance Board, Walking Heel-to-Toe Forwards, and Hopping on Mats, resulted in good reliability, except for the Drawing a Trail, which showed moderate reliability. The results of this study were similar to that of Valentini et al.'s study of 43 typical children, aged 6 to 11 years, showing good reliability (ICC = 0.86-0.99) [11]. However, a Norwegian study of 30 typical children, aged 7-9 years, recorded poor to moderate interrater reliability (ICC = 0.39-0.68) [3]. The difference between this study in Thailand and that in Norway is a variation in how the tests were administrated. The study in Norway used two testers to administer the test to each child twice, separately on the same day, while this study in Thailand tested each child once, with one tester scoring and a second tester observing and scoring. The method used in the Norway study might be affected by the motor learning, motivation, and attention of the child when he/she had to perform the same activities 2-4 times within a day. However, the procedure of this study followed that of Portney and Watkins, who mentioned that the best way to established interrater reliability is when all raters can measure a response during a single test and observe a subject simultaneously and independently [19]. However, the Drawing a Trail was the only item that showed moderate reliability. This might be caused by the instructions given and bias of the testers. According to the instructions, if a child could complete the first trial without errors, then he/she was not tested for the second trial. However, the testers would determine whether the second trial was necessary. Some testers administered this item twice, while another tested once or twice. Duncan stated that characteristics of the testers such as strictness or leniency had an influence on the scores [20]. Therefore, instructions for the test item should be adjusted so that the testers administer it in the same way.

## 5. Implication for Clinicians

The results of the psychometric property study imply that the Thai-MABC2-AB2 can be used to examine movement difficulty in Thai children, measuring a child's progress and an outcome of intervention program.

## 6. Recommendations for Future Research

This study was an initial step for exploring the psychometric properties of the Thai-MABC2-AB2 before using this standardized test to detect movement difficulty in Thailand. Future studies should examine other psychometric properties, for example, test-retest reliability, and construct validity of the Thai-MABC2-AB2. In addition, the motor performance norm for Thai children should be also established.

## 7. Conclusion

This study revealed that the Thai-MABC2-AB2 is valid for examining movement difficulty. The results from testers were consistent. Therefore, the Thai-MABC2-AB2 is appropriated for use in the Thai context and can be used for detecting movement difficulty in Thai children.

## Figures and Tables

**Table 1:** The results of content validity in the IOC index in each test.

MABC-2 tests	Language clarity	Language pertinence
Manual Dexterity	0.73	0.83
Aiming and Catching	0.88	0.95
Balance	0.89	0.94

**Table 2:** Statistical results of inter-rater reliability, using raw scores.

MABC-2 subtests	Tester 1 (*n* = 29)	Tester 2 (*n* = 29)	ICC	95% CI
Manual Dexterity				
Placing Pegs (preferred hand) (sec)	23.76 ± 2.68	23.86 ± 2.68	0.99	0.98-0.99
Placing Pegs (nonpreferred hand) (sec)	26.55 ± 3.97	26.62 ± 4.05	0.99	0.99-0.99
Threading Lace (sec)	18.28 ± 3.34	18.48 ± 3.23	0.99	0.99-0.99
Drawing a Trail (no. of errors)	0.52 ± 0.68	0.41 ± 0.73	0.71	0.38-0.86
Aiming and Catching				
Catching with Two Hands (no. of catches)	5.45 ± 2.94	5.55 ± 2.87	0.99	0.98-0.99
Throwing a Beanbag onto a Mat (no. of hits)	6.62 ± 1.54	6.62 ± 1.59	0.94	0.88-0.97
Balance				
One-Balance Board (other leg) (sec)	22.31 ± 8.86	22.34 ± 8.89	1.00	0.99-1.00
Walking Heel-to-Toe Forwards (no. of steps)	13.45 ± 3.41	13.52 ± 3.29	0.99	0.99-0.99
Hopping on Mats (best leg) (no. of hops)	4.97 ± 0.18	5.00 ± 0.00	-^a^	-^a^
Hopping on Mats (other leg) (no. of hops)	4.48 ± 0.63	4.41 ± 0.73	0.96	0.92-0.98

-^a^No variation of data; ICC could not be calculated.

## Data Availability

The data used to support the findings of this study are available from the corresponding author upon request.
